# Supplementary material for: Light quality-regulated anthocyanin biosynthesis in Lilium leichtlinii subsp. maximowiczii bulbs: A multi-omics perspective
Source: PLoS One. 2026 May 13;21(5):e0347686. doi: 10.1371/journal.pone.0347686 (PMC13170870; doi:10.1371/journal.pone.0347686)
Supplement: S1 Table — 1. Primers for verification of L-bulb transcriptome by qRT-PCR; 2. The qRT-PCR reaction system; 3. for qRT-PCR reaction procedures; 4. Second-class classification of the metabolites; 5. Statistical Table of KEGG Pathways (log2FC > 4.79); 6. Statistical table of the assembly results; 7. annotation statistics. (DOCX) [file pone.0347686.s006.docx]

1. Primers for verification of L-bulb transcriptome by qRT-PCR

| Primers | Primer Sequences (5'-3') | Time (℃) | Product length  （bp） |
| --- | --- | --- | --- |
| CHS2（Cluster-27448.10）-F | CTACGACTTCATCCGCCTCC | 62 | 153 |
| CHS2（Cluster-27448.10）-R | TCCGATGAGACAACCAGCAC | 60 |  |
| CHS3（Cluster-6173.4）-F | GTCCGATCCCGACACTTCTG | 62 | 90 |
| CHS3（Cluster-6173.4）-R | TCAATGGCACCCTCCGAATC | 60 |  |
| CHS1（Cluster-6173.2）-F | ATCAGAGAGCCACCTCGACA | 60 | 104 |
| CHS1（Cluster-6173.2）-R | GAGGTCGCTCCACAGAAGTG | 60 |  |
| CHSC-2（Cluster-2480.9）-F | TTGTCGAGGTCCCAAAGCTC | 60 | 198 |
| CHSC-2（Cluster-2480.9）-R | AGCAGCCCTGCTGGTACATC | 60 |  |
| CHSC-1（Cluster-2480.6）-F | TGGGCCATCAGAATCACACC | 60 | 84 |
| CHSC-1（Cluster-2480.6）-R | TCAGAGCCGACGATAACAGC | 60 |  |
| CHS2-2（Cluster-3851.0）-F | TCGCTGGTTCTGAAGGTGAC | 60 | 239 |
| CHS2-2（Cluster-3851.0）-R | TGTGCGCAACTTCTCTTTGC | 60 |  |
| CHI-1（Cluster-7233.0）-F | ACTGTCCGTCAGGAGGAGAA | 60 | 248 |
| CHI-1（Cluster-7233.0）-R | CACCACGAGCCTAACTGTCA | 60 |  |
| CHI-2（Cluster-21843.0）-F | ATTGGGCCATGGTCTCACTG | 60 | 135 |
| CHI-2（Cluster-21843.0）-R | TCAGCGAGCTCTGCGCCTTT | 58 |  |
| CHI-3（Cluster-24322.0）-F | TCGCGTATCCGAAGTCGAAC | 60 | 111 |
| CHI-3（Cluster-24322.0）-R | CTCCCCGATCAACAAGCAGA | 60 |  |
| CHIa（Cluster-28544.0）-F | GGGGTCGATCATCGGAGAAC | 62 |  |
| CHIa（Cluster-28544.0）-R | AGAAGCTCGGAAATGCGGAA | 58 | 75 |
| DFR（Cluster-2527.10）-F | ACCACGCCAAACACAAGAGA | 58 | 139 |
| DFR（Cluster-2527.10）-R | TTGTTCATTGTGCCCTCTCA | 58 |  |

1. Primers for verification of L-bulb transcriptome by qRT-PCR

| Primers | Primer Sequences (5'-3') | Time (℃) | Product length  （bp） |
| --- | --- | --- | --- |
| F3H（Cluster-24939.0）-F | GGACGAGGATGGACCGATTC | 60 | 213 |
| F3H（Cluster-24939.0）-R | CTGGAGACGATGAAGCCTCC | 62 |  |
| F3`H（Cluster-27140.0）-F | TGAGCTCGCCGGAAAAGATT | 58 | 86 |
| F3`H（Cluster-27140.0）-R | CAGTGTCGGTCAGTTTCCCA | 60 |  |
| FLS-2（Cluster-1772.0）-F | TCTTCGAGGAGGTACCCCAG | 62 | 155 |
| FLS-2（Cluster-1772.0）-R | CCTCTTCTCCGGCCAAACAT | 60 |  |
| FLS-1（Cluster-17722.0）-F | TCCAAAGAGAGCATCTCCGC | 60 | 115 |
| FLS-1（Cluster-17722.0）-R | AGTTTAGCGGCCTCGTCATC | 60 |  |
| FLS-3（Cluster-13096.0）-F | CAACCCCAAGAGTGACCTCC | 62 | 77 |
| FLS-3（Cluster-13096.0）-R | TCGATCGATATAACGAGGGT | 60 |  |
| FLS-4（Cluster-16697.0）-F | GGCGATCCAATTCCCGATCT | 62 | 229 |
| FLS-4（Cluster-16697.0）-R | TCCAATCAAGCACTCCGTCC | 60 |  |
| ANS（Cluster-22832.0）-F | GCAGCTTGAGTGGGAGGATT | 60 | 146 |
| ANS（Cluster-22832.0）-R | GCATCTTGGTCACCACCACT | 60 |  |
| ANR-1（Cluster-21494.0）-F | GTGCCCAGTATAAGGATGGG | 62 | 149 |
| ANR-1（Cluster-21494.0）-R | GCAGCTTTGGTCTCAGCAAC | 60 |  |
| ANR-2（Cluster-21738.0）-F | GGGGATGCACTGATTCCACAT | 60 | 123 |
| ANR-2（Cluster-21738.0）-R | CAGAGGCAACGAGCCTAACA | 60 |  |
| ANR-3（Cluster-21738.2）-F | TTGTGAAAGGTCTGGGTCCG | 60 | 152 |
| ANR-3（Cluster-21738.2）-R | TCTCCTCGCAGTGTGCAAAT | 58 |  |

| FG2（Cluster-30829.0）-F | TCAAACAAGCCCTCGACCTC | 60 | 76 |
| --- | --- | --- | --- |
| FG2（Cluster-30829.0）-R | TGGAAGACGAGTTGGGGACG | 60 |  |

1. Primers for verification of L-bulb transcriptome by qRT-PCR

| Primers | Primer Sequences (5'-3') | Time (℃) | Product length  （bp） |
| --- | --- | --- | --- |
| BZ1-1（Cluster-21866.1）-F | ACGGCTTCATTGAACGGACT | 58 |  |
| BZ1-1（Cluster-21866.1）-R | GCCATCATGTGGTCTCCGAA | 60 | 187 |
| 3GT（Cluster-5566.0）-F | GGGATCAGGCGGAATAAGGG | 62 | 128 |
| 3GT（Cluster-5566.0）-R | CAGCAAGGCTGTAAGTCCCA | 60 |  |
| BZ1-2（Cluster-30886.5）-F | CACTCCTCCGGACTTTGGTC | 60 | 219 |
| BZ1-2（Cluster-30886.5）-R | TCCGGTATGGCAGAGATG | 60 |  |
| Actin-F | GCATCACACCTTCTACAACG | 58 |  |
| Actin-R | GAAGAGCATAACCCTCATAGA | 56 |  |
| 18S-F | CGTTTCGGGCATGATTTGTGG | 60 |  |
| 18S-R | TCGCATTTCGCTACGTTCTTC | 58 | —— |

2. The qRT-PCR reaction system

| Reaction system | |
| --- | --- |
| 2×Taq SYBR Green qPCR Mix | 12.5 μl |
| Forward Primer | 0.5 μl |
| Reverse Primer | 0.5 μl |
| cDNA | 1 μl |
| ddH2O up to | 25 μl |

3. for qRT-PCR reaction procedures

| Step | Temperature(℃) | Time | Cycles |
| --- | --- | --- | --- |
| Initial Denaturation | 94 ℃ | 2 min | 1 |
| Denaturation | 94 ℃ | 10 s | 40 |
| Annealing | 60 ℃ | 30 s | 40 |
| Extension | 72 ℃ | 20 s | 40 |

4. Second-class classification of the metabolites

| Class I | Class II | Number | Total |
| --- | --- | --- | --- |
| Flavonoids | Flavonols | 96 | 252 |
|  | Flavones | 44 |  |
|  | Flavanones | 26 |  |
|  | Other Flavonoids | 23 |  |
|  | Anthocyanidins | 22 |  |
|  | Flavanols | 14 |  |
|  | Flavanonols | 10 |  |
|  | Chalcones | 9 |  |
|  | Isoflavones | 5 |  |
|  | Tannin | 3 |  |

5. Statistical Table of KEGG Pathways (log2FC> 4.79)

Note: * denotes structurally similar isomers of the compound.

| Class II | Number | Index | Compounds | Formula | Type | log_2_FC |
| --- | --- | --- | --- | --- | --- | --- |
| Flavonols | 30 | Lmsp003252 | Isorhamnetin-3-O-sophoroside | C_28_H_32_O_17_ | up | 9.65 |
|  |  | mws0091 | Quercetin-3-O-glucoside (Isoquercitrin) | C_21_H_20_O_12_ | up | 9.49 |
|  |  | mws0061 | Quercetin-3-O-galactoside (Hyperin) | C_21_H_20_O_12_ | up | 9.13 |
|  |  | mws1329 | Quercetin-7-O-glucoside | C_21_H_20_O_12_ | up | 8.88 |
|  |  | mws0856 | Quercetin-4'-O-glucoside (Spiraeoside)* | C_21_H_20_O_12_ | up | 8.54 |
|  |  | pma0214 | 6-C-Methylquercetin-3-O-glucoside | C_22_H_22_O_12_ | up | 8.34 |
|  |  | Lmdp003286 | Quercetin-3-O-alloside; Isohyperoside* | C_21_H_20_O_12_ | up | 8.33 |
|  |  | Lmjp003295 | 6-Methoxykaempferol-3-O-glucoside* | C_22_H_22_O_12_ | up | 8.27 |
|  |  | Smgp004575 | Quercetin-5-O-β-D-glucoside* | C_21_H_20_O_12_ | up | 8.18 |
|  |  | pmp001309 | 6-Hydroxykaempferol-7-O-glucoside | C_21_H_20_O_12_ | up | 8.10 |
|  |  | Zbpp001992 | Isorhamnetin-3-O-Glucoside* | C_22_H_22_O_12_ | up | 7.77 |
|  |  | Lmjp002906 | Rhamnetin-3-O-Glucoside* | C_22_H_22_O_12_ | up | 7.57 |
|  |  | pmp000589 | Quercetin-7-O-(6''-malonyl)glucoside | C_24_H_22_O_15_ | up | 7.35 |
|  |  | mws0059 | Quercetin-3-O-rutinoside (Rutin) | C_27_H_30_O_16_ | up | 7.21 |
|  |  | Zmhp004034 | Gossypetin-7-O-rhamnoside; Rhodiolgin | C_21_H_20_O_12_ | up | 6.99 |
|  |  | Zmhp005139 | Tamarixetin-3-O-(6''-malonyl)glucoside* | C_25_H_24_O_15_ | up | 6.94 |
|  |  | pmn001583 | Quercetin-3-O-robinobioside | C_27_H_30_O_16_ | up | 6.88 |

5. Statistical Table of KEGG Pathways (log2FC> 4.79)

Note: * denotes structurally similar isomers of the compound.

| Class II | Number | Index | Compounds | Formula | Type | log_2_FC |
| --- | --- | --- | --- | --- | --- | --- |
|  |  | Zbpp002027 | Isorhamnetin-3-O-(6''-malonyl)glucoside* | C_25_H_24_O_15_ | up | 6.83 |
|  |  | Zbsp004301 | Quercetin-7-O-rutinoside* | C_27_H_30_O_16_ | up | 6.77 |
|  |  | Lmsp004166 | Quercetin-3-O-glucoside-7-O-rhamnoside* | C_27_H_30_O_16_ | up | 6.63 |
|  |  | Lmjp002461 | Quercetin-3-O-neohesperidoside* | C_27_H_30_O_16_ | up | 6.48 |
|  |  | Lmmp003091 | Quercetin-3-O-(4''-O-glucosyl)rhamnoside* | C_27_H_30_O_16_ | up | 6.44 |
|  |  | Lmsp004045 | Isorhamnetin-3-O-glucoside-7-O-rhamnoside | C_28_H_32_O_16_ | up | 5.40 |
|  |  | Zblp004717 | 3'-methoxyquercetin-3-O-L-rhamnosyl(1→2)-glucopyranoside* | C_28_H_32_O_16_ | up | 5.23 |
|  |  | Lmdp002969 | Myricetin-3-O-galactoside* | C_21_H_20_O_13_ | up | 5.06 |
|  |  | Lmsp004721 | Tamarixetin-3-O-glucoside-7-O-rhamnoside* | C_28_H_32_O_16_ | up | 5.02 |
|  |  | Lmfp003403 | Quercetagetin-7-O-glucoside(Quercetagitrin)* | C_21_H_20_O_13_ | up | 5.02 |
|  |  | Lmfn004065 | Morin-3-O-arabinoside* | C_20_H_18_O_11_ | up | 4.93 |
|  |  | Lmpp003465 | Myricetin-3-O-β-D-glucoside* | C_20_H_18_O_11_ | up | 4.80 |
|  |  | Lmmp001947 | Gossypetin-3-O-glucoside* | C_21_H_20_O_13_ | up | 4.79 |
| Flavones | 4 | Cmhp002692 | Madreselvin A | C_28_H_32_O_17_ | up | 9.31 |
|  |  | Hmcn001884 | 6-Hydroxyluteolin 5-glucoside* | C_21_H_20_O_12_ | up | 8.67 |
|  |  | Hmgp002036 | Nepetin-7-O-glucoside(Nepitrin)* | C_22_H_22_O_12_ | up | 8.22 |

5. Statistical Table of KEGG Pathways (log2FC> 4.79)

Note: * denotes structurally similar isomers of the compound.

| Class II | Number | Index | Compounds | Formula | Type | log_2_FC |
| --- | --- | --- | --- | --- | --- | --- |
|  |  | pmb3012 | Chrysoeriol-7-O-glucoside | C_22_H_22_O_11_ | up | 7.86 |
| Flavanones | 6 | pme1598 | Hesperetin-5-O-glucoside | C_22_H_24_O_11_ | up | 8.66 |
|  |  | Wahp004841 | Homoeriodictyol-7-O-β-O-glucoside* | C_22_H_24_O_11_ | up | 8.34 |
|  |  | Zbhp004510 | Hesperetin-3'-O-glucoside* | C_22_H_24_O_11_ | up | 7.97 |
|  |  | MWS20145 | Eriodictyol-7-O-glucoside* | C_21_H_22_O_11_ | up | 5.24 |
|  |  | Zbnp004170 | Eriodictyol-3'-O-glucoside* | C_21_H_22_O_11_ | up | 5.17 |
|  |  | HJN087 | Naringenin-4'-O-glucoside* | C_21_H_22_O_10_ | up | 5.05 |
| Other Flavonoids | 2 | Wbtn004512 | 1,8-dihydroxy-4,5-dimethoxy-3-{[(2s,3r,4s,5s,6r)-3,4,5-trihydroxy-6-(hydroxymethyl)oxan-2-yl]oxy}xanthen-9-one | C_21_H_22_O_12_ | up | 7.76 |
|  |  | Wbtn004861 | 1,8-dihydroxy-2,6-dimethoxy-5-{[(2s,3r,4s,5s,6r)-3,4,5-trihydroxy-6-(hydroxymethyl)oxan-2-yl]oxy}xanthen-9-one | C_21_H_22_O_12_ | up | 7.43 |
| Anthocyanidins | 6 | Zbcp002823 | Cyanidin-3-O-rutinoside (Keracyanin) | C_27_H_31_O_15+_ | up | 8.31 |
|  |  | Zblp102242 | Cyanidin-3-O-galactoside* | C_21_H_21_O_11+_ | up | 7.16 |
|  |  | Zblp002068 | Cyanidin-3-O-glucoside* | C_21_H_21_O_11+_ | up | 7.12 |
|  |  | Zblp001862 | Delphinidin-3-O-galactoside | C_21_H_21_O_12+_ | up | 6.14 |

5. Statistical Table of KEGG Pathways (log2FC> 4.79)

Note: * denotes structurally similar isomers of the compound.

| Class II | Number | Index | Compounds | Formula | Type | log_2_FC |
| --- | --- | --- | --- | --- | --- | --- |
|  |  | Lmpp003815 | Petunidin-3-O-(6''-O-p-Coumaroyl)glucoside | C_31_H_29_O_14+_ | up | 5.50 |
|  |  | pme1398 | Delphinidin-3-O-glucoside (Mirtillin) | C_21_H_21_O_12+_ | up | 5.47 |
| Flavanonols | 2 | mws0044 | Taxifolin(Dihydroquercetin) | C_15_H_12_O_7_ | up | 8.11 |
|  |  | Lmlp005236 | Dihydrokaempferol-3-O-glucoside* | C_21_H_22_O_11_ | up | 4.97 |

6. Statistical table of the assembly results

| Type | Number | Mean Length | N50 | N90 |
| --- | --- | --- | --- | --- |
| Transcript | 117922 | 970 | 1478 | 422 |
| Unigene | 62523 | 1199 | 1603 | 583 |

7. annotation statistics

| Database | Number of Genes | Percentage (%) |
| --- | --- | --- |
| KEGG | 33129 | 52.99 |
| Nr | 44018 | 70.40 |
| SwissProt | 33997 | 54.38 |
| TrEMBL | 43933 | 70.27 |
| KOG | 26165 | 41.85 |
| GO | 37672 | 60.25 |
| Pfam | 33498 | 53.58 |
| Annotated in at least one Database | 45033 | 72.03 |
| Total Unigenes | 62523 | 100.00 |
