# Supplementary material for: Light quality-regulated anthocyanin biosynthesis in Lilium leichtlinii subsp. maximowiczii bulbs: A multi-omics perspective
Source: PLoS One. 2026 May 13;21(5):e0347686. doi: 10.1371/journal.pone.0347686 (PMC13170870; doi:10.1371/journal.pone.0347686)
Supplement: Attachment — Submitted filename: 20260227Review Comments.docx [file pone.0347686.s007.docx]

The paper provides a comprehensive investigation of the regulation of anthocyanins in lilies through omics approaches and contains a substantial amount of information. However, the experimental design with regard to temperature requires refinement. The paper is acceptable overall, and minor revisions are recommended for acceptance.

1. References to research on *Lilium leichtlinii* var. *maximowiczii* should be added between lines 68 and 73. For instance, it would be beneficial to readers if its medicinal value were clarified.
2. The word 'bulbs' does not require italics on line 93.
3. Line98&Line107. Please explain why a temperature of 10°C was selected for the light treatment and why 4°C was chosen as one of the three temperatures. What considerations underlie these decisions?
4. Line 140: Freeze-dried lilies should be ground into powder while they are still under liquid nitrogen conditions.
5. How should one explain the fact that metabolic analysis only uses samples from the six-day treatment period? And why is this the case?
6. Line 177: The thermo instruments should be labelled with their model and the country of manufacture.
7. The cDNA reverse transcription step should be included on line 188.
8. The use of the internal reference gene should be supplemented on line 196.
9. What is the explanation for the build-up of anthocyanins at higher temperatures, yet the observation that the effect at 37°C is less marked than at 25°C?
10. In conclusion, line 545 is best applied to edible lilies.
