# Supplementary material for: Light quality-regulated anthocyanin biosynthesis in Lilium leichtlinii subsp. maximowiczii bulbs: A multi-omics perspective
Source: PLoS One. 2026 May 13;21(5):e0347686. doi: 10.1371/journal.pone.0347686 (PMC13170870; doi:10.1371/journal.pone.0347686)
Supplement: Attachment — Submitted filename: Response to Reviewers.docx [file pone.0347686.s009.docx]

**Response letter**

Dear Editor,

We would like to thank you for giving us a chance to resubmit the paper, and thank the reviewers for giving us constructive suggestions which have helped substantiate the conclusions. We have addressed all the issues in the following ways: revising the manuscript content based on the reviewers’ comments, providing clarifications, or presenting supplementary results. Here we submit a new version of our manuscript with the title “**Light Quality-Regulated Anthocyanin Biosynthesis in *Lilium leichtlinii* subsp. *maximowiczii* Bulbs: A Multi-Omics Perspective**”, which has been modified according to the reviewers’ suggestions. We mark all the changes in red in the revised manuscript. Point-by-point responses to the reviewer’s comments are given below, with the reviewer’s comments given verbatim in plain Italic type, and our responses in Regular:

*Journal Requirements:*

*When submitting your revision, we need you to address these additional requirements.*

*1. Please ensure that your manuscript meets PLOS ONE's style requirements, including those for file naming. The PLOS ONE style templates can be found at*

*[https://journals.plos.org/plosone/s/file?id=wjVg/PLOSOne_formatting_sample_main_body.pdf](https://journals.plos.org/plosone/s/file?id=wjVg/PLOSOne_formatting_sample_main_body.pdf) and [https://journals.plos.org/plosone/s/file?id=ba62/PLOSOne_formatting_sample_title_authors_affiliations.pdf](https://track.editorialmanager.com/CL0/https://journals.plos.org/plosone/s/file?id=ba62/PLOSOne_formatting_sample_title_authors_affiliations.pdf/1/010f019cad9cf22b-107cd4e0-730d-44db-9790-39358d8f8a87-000000/mKRIbAOc2YrGHH_CWkLIYnO3io_lDBNQfcMjmf16h10=252)*

**Response**: Thank you for your reminder. We have carefully revised the manuscript and strictly formatted the full text in accordance with the PLOS ONE style template, including the title page, author information, main text, reference formatting, and have ensured compliance with all file naming conventions.

2. *In your Methods section, please provide additional information regarding the permits you obtained for the work. Please ensure you have included the full name of the authority that approved the field site access and, if no permits were required, a brief statement explaining why.*

**Response**: Thank you for your reminder. This study did not involve any fieldwork requiring permits: all data were obtained from laboratory experiments, and the experimental samples were shipped by the seller via courier. No field sampling or access to regulated areas was conducted. Therefore, no fieldwork permits were required.

*3. We note that the grant information you provided in the ‘Funding Information’ and ‘Financial Disclosure’ sections do not match. When you resubmit, please ensure that you provide the correct grant numbers for the awards you received for your study in the ‘Funding Information’ section.*

**Response**: Thank you for your reminder. Upon verification, it is confirmed that this study was supported by the Science and Technology Development Program of Jilin Province (2024JB427L35).

*4. Thank you for stating the following financial disclosure:*

*“This study was supported by Science and Technology Development Program of Jilin Province (2024JB427L35).” Please state what role the funders took in the study. If the funders had no role, please state: "The funders had no role in study design, data collection and analysis, decision to publish, or preparation of the manuscript." If this statement is not correct you must amend it as needed. Please include this amended Role of Funder statement in your cover letter; we will change the online submission form on your behalf.*

**Response**: Thank you for pointing this out. We have confirmed: "The funders had no role in study design, data collection and analysis, decision to publish, or preparation of the manuscript." This statement has been included in the cover letter.

5. *Please note that funding information should not appear in any section or other areas of your manuscript. We will only publish funding information present in the Funding Statement section of the online submission form. Please remove any funding-related text from the manuscript.*

**Response**: Thank you for your reminder. We have removed all funding-related text from the main body of the manuscript.

6. *In the online submission form, you indicated that your data is available only on request from a third party. Please note that your Data Availability Statement is currently missing [the name of the third party contact or institution / contact details for the third party, such as an email address or a link to where data requests can be made]. Please update your statement with the missing information.*

**Response**: Thank you for your reminder. We have supplemented the missing contact information as required.

7. *Please amend the manuscript submission data (via Edit Submission) to include author “Shuai Li , Cheng Wang, Bingyan Liu , Qi Wu, Yanli Ma, Xinzhu Dai, Yuanhang Zhou, Xinhai Yu, Tianliang Wang”.*

**Response**: Thank you for your reminder. We have added the following authors to the author list: *Shuai Li , Chen Wang, Bingyan Liu , Qi Wu, Yanli Ma, Xinzhu Dai, Yuanhang Zhou, Xinhai Yu, Tianliang Wang.*

8. *We notice that your supplementary figures are uploaded with the file type 'Figure'. Please amend the file type to 'Supporting Information'. Please ensure that each Supporting Information file has a legend listed in the manuscript after the references list.*

**Response**: Thank you for your reminder. The supplementary data originally uploaded as "Figure" type have now been reclassified as "Supporting Information" type. Additionally, we have ensured that each supporting information file has a corresponding legend listed after the reference list, in compliance with PLOS ONE's specifications.

9. *If the reviewer comments include a recommendation to cite specific previously published works, please review and evaluate these publications to determine whether they are relevant and should be cited. There is no requirement to cite these works unless the editor has indicated otherwise.*

**Response**: Thank you for the reviewers' valuable suggestion. We confirm that the newly added references are relevant to this study and have not been previously cited.

*Additional Editor Comments:
Both reviewers noticed that the methodology requires more detailed description in several specific points. Please mind that Lilium leichtlinii subsp. maximowiczii (Regel) J.Compton is a subspecies, not a variety (see e.g., [https://powo.science.kew.org/taxon/urn:lsid:ipni.org:names:77215715-1](https://powo.science.kew.org/taxon/urn:lsid:ipni.org:names:77215715-1)).*

**Response**: Thank you for pointing out that *Lilium leichtlinii* subsp. *maximowiczii* (Regel) J.Compton should be recognized as a subspecies rather than a variety, and for providing the reference link to the Kew database. We have revised the taxonomic description accordingly in the relevant section of the manuscript and have verified the use of related terms to ensure accuracy in taxonomic designation.

*"Lilium spp." cannot contain 155 species, but the genus Lilium can contain them. "Lilium" in italics (e.g. L48). "unicolor" in italics (L63).*

**Response**: Thank you for your careful correction regarding the usage of the genus Lilium. We have amended the inappropriate expressions such as “*Lilium spp*.” to “*Lilium*” and have italicized all Latin names, including *Lilium* (line 44) and *unicolor* (line 68), to comply with nomenclature standards.

*I suggest dividing Figure 5 into several, more easily readable figures, since the font size in D and E is barely visible.*

**Response**: Thank you for your suggestion regarding the readability of Figure 5. As recommended, we have divided the original figure into several separate figures and adjusted the font size to ensure that all information is clearly presented and easily interpretable for readers.

*The authors are strongly encouraged to have the manuscript proofread by a native English speaker or a professional editing agency to polish the text and improve general readability.*

**Response**: Thank you for your advice on language quality. We have engaged a native English speaker and a professional editing service to proofread the entire manuscript, aiming to refine the language and improve overall readability.

**Reviewer: 1**

*1. The manuscript reports large fold changes (e.g., cyanidin-3-rutinoside FC = 316.53, DFR FPKM = 1425.78) (line no. 402) that appear unusually high; ensure normalization and log-transformation are appropriately applied.*

**Response**: We thank the reviewer for raising this concern. Regarding the large fold changes reported in the manuscript, we have re-examined the procedures for data normalization and log transformation. The verification results confirm that these high fold changes truly reflect significant differences in the expression levels of the corresponding metabolites and genes between the treatment and control groups, with consistent measurements across biological replicates.

2. *De-italicize Bulb to Bulb wherever necessary.*

**Response**: Thank you for your careful review. As suggested, we have removed the italics from “Bulb” throughout the manuscript to comply with standard formatting guidelines (line 100).

3. *The Introduction section contains several sentences written in the future tense (e.g., “This study will elucidate…”). Please revise these to present tense to accurately reflect completed research and maintain consistency with standard scientific writing conventions.*

**Response**: We thank the reviewer for the careful reading. As suggested, we have changed the tense in the Introduction section from future to present tense to better conform to standard academic writing conventions. For example, in the revised manuscript, "This study will systematically elucidate" has been changed to "This study systematically elucidates" (Line 84). All changes have been highlighted in the revised manuscript.

4. *Section 2.1.2: The methodology requires more detailed description—please include specific temperature control conditions (e.g., type of incubator, monitoring frequency, humidity). Additionally, spell out the abbreviations Dc and WLc in full when they first appear, and consider removing or minimizing abbreviations throughout this section for improved readability and clarity.*

**Response**: We thank the reviewer for their valuable suggestions. Accordingly, we have supplemented Section 2.1.2 with specific details on the temperature control conditions, including the equipment used and the humidity control range. In addition, to enhance readability and clarity, we have spelled out the abbreviations “Dc” and “WLc” in full upon their first appearance in the text (lines 106–108).

5. *Kindly clarify whether the samples used for RNA seq, q-RT PCR and metabolomics were taken from same batch or different batch and also mention clearly the stages of sample preparation for the same.*

**Response**:We thank the reviewer for raising this critical point regarding sample consistency. To clarify, the samples used for RNA sequencing, qRT-PCR, and metabolomics analysis were all derived from the same batch of experiments. Specifically, all samples were subjected to identical treatment conditions to ensure comparability across the different omics datasets. This information has now been added in Section 2.6.

6. *Clearly mention the number of replicates taken to perform transcriptomics, metabolomics and qRT-PCR in the material and methods section.*

**Response**: Thank you for pointing this out. In the transcriptomics, metabolomics, and qRT‑PCR experiments, three technical replicates were set for each sample to ensure reproducibility and reliability. The corresponding replicate numbers have now been added to the Materials and Methods section.

7. *The font size of figures should be improved to enhance readability and clarity and include bootstrap values in the phylogenetic tree.*

**Response**: Thank you for your valuable comments. We have improved the font size of the figures to enhance readability and clarity, and we have also included bootstrap values in the phylogenetic tree.

*8. Line number 310 word “Ctrl” has not mentioned anywhere in the material and method section.*

**Response**: Thank you for your insightful comment. In the manuscript, “Ctrl” denotes the control group used in the experiments. To address this concern, we have now added a clarification in the Materials and Methods section (line 164) to ensure that readers can clearly understand this abbreviation. We appreciate your valuable suggestion, which has helped improve the clarity and rigor of our work.

**Reviewer: 2**

*Comments to the Author*

*The paper provides a comprehensive investigation of the regulation of anthocyanins in lilies through omics approaches and contains a substantial amount of information. However, the experimental design with regard to temperature requires refinement. The paper is acceptable overall, and minor revisions are recommended for acceptance.*

**Major Revisions**
*1. References to research on Lilium leichtlinii var. maximowiczii should be added between lines 68 and 73. For instance, it would be beneficial to readers if its medicinal value were clarified.*

**Response**: Thank you very much for your valuable suggestion. We have added relevant references regarding Lilium leichtlinii var. maximowiczii between lines 78 and 79 to highlight its potential medicinal value and enhance readers' understanding of the significance of this species.

1.	Wang, Y., et al., *Traditional uses, nutritional properties, phytochemical metabolites, pharmacological properties, and potential applications of Lilium spp.: a systematic review.* Frontiers in Pharmacology, 2025. **16**: p. 1713957.

*2. The word 'bulbs' does not require italics on line 93.*

**Response**: Thank you for your careful review. As suggested, we have removed the italics from “Bulb” throughout the manuscript to comply with standard formatting guidelines （line 100）.

*3. Line98&Line107. Please explain why a temperature of 10°C was selected for the light treatment and why 4°C was chosen as one of the three temperatures. What considerations underlie these decisions?*

**Response**: We thank the reviewer for raising this question. The temperature of 10°C was selected because it represents a common critical temperature during plant growth or postharvest storage. For crops such as edible lily, this temperature effectively extends the shelf life and delays senescence without causing significant chilling injury, thereby facilitating the observation of the independent effects of light on color regulation. This approach helps ensure that the observed physiological changes can be primarily attributed to the light treatment itself, rather than to damage caused by low temperature.

The temperature of 4°C was chosen as it represents a standard condition for low-temperature stress or chilling injury. By including this temperature, we can clearly characterize the physiological responses of edible lily under extreme low-temperature stress, such as whether anthocyanins accumulate or degrade abnormally in response to such stress.

*4. Line 140: Freeze-dried lilies should be ground into powder while they are still under liquid nitrogen conditions.*

**Response**: Thank you for your valuable comment. We confirm that the freeze-dried lily samples were indeed ground into powder under liquid nitrogen conditions. This information was inadvertently omitted in the original manuscript and has now been added at line 147 in the revised version. We appreciate your careful review and the opportunity to improve our manuscript.

*5. How should one explain the fact that metabolic analysis only uses samples from the six-day treatment period? And why is this the case?*

**Response**: Thank you for your insightful question regarding the selection of samples from the 6-day treatment period for metabolomic analysis. Our decision was based on experimental observations and quality assessments. Specifically, we observed a distinct color change in the lily bulbs on day 6, and the overall quality at this time point was superior to that of the day 9 and day 12 samples. Therefore, day 6 was considered the most representative time point for capturing key metabolic changes associated with the treatment.  *6. Line 177: The thermo instruments should be labelled with their model and the country of manufacture.*

**Response**: Thank you for your valuable comment. We have now specified the model and country of manufacture for the thermodynamic instrument at line 187 in the revised manuscript to ensure completeness and compliance with journal requirements. We appreciate your attention to detail, which has helped improve the clarity and rigor of our work. *7. The cDNA reverse transcription step should be included on line 188.*

**Response**: We thank the reviewer for the correction. We have now supplemented the description of the cDNA reverse transcription step at line 203. *8. The use of the internal reference gene should be supplemented on line 196.*

**Response**: We thank the reviewer for the suggestion. We have now included the information regarding the internal reference gene used at line 210.

*9. What is the explanation for the build-up of anthocyanins at higher temperatures, yet the observation that the effect at 37 °C is less marked than at 25 °C?*

**Response**: We thank the reviewer for this comment. Regarding the question of why anthocyanin accumulation was less pronounced at 37 *°C* compared to 25 *°C*, we have provided some analysis in the Discussion section. We attribute this phenomenon primarily to the thermal inactivation of anthocyanin biosynthetic enzymes. The biosynthesis of anthocyanins requires the involvement of a series of enzymes, such as phenylalanine ammonia-lyase (PAL). The optimal temperature range for most of these synthetases is around 25 *°C*, where their catalytic efficiency is highest. When the temperature rises to 37 *°C*, it approaches or exceeds the thermal inactivation threshold for some key enzymes, leading to a decline in the overall biosynthesis rate.

1.	Jaakola, L., *New insights into the regulation of anthocyanin biosynthesis in fruits.* Trends in plant science, 2013. **18**(9): p. 477-483.

*10. In conclusion, line 545 is best applied to edible lilies.*

**Response**: We thank the reviewer for the valuable suggestion. In response, we have revised the manuscript accordingly, with the modification clearly indicated at line 650. Considering the context involving postharvest technologies, we determined that using "edible lily" more accurately reflects the specific characteristics of the plant material studied compared to the more general term "lily." Therefore, we have replaced "lily" with "edible lily" in the text. We appreciate the reviewer's help in enhancing the precision of our manuscript.
